# Diversity of *Bartonella* spp. in Bats, Southern Vietnam

**DOI:** 10.3201/eid2107.141760

**Published:** 2015-07

**Authors:** Pham Hong Anh, Nguyen Van Cuong, Nguyen Truong Son, Ngo Tri Tue, Michael Kosoy, Mark E.J. Woolhouse, Stephen Baker, Juliet E. Bryant, Guy Thwaites, Juan J. Carrique-Mas, Maia A. Rabaa

**Affiliations:** Oxford University, Ho Chi Minh City, Vietnam (P.H. Anh, N. Van Cuong, N.T. Son, N.T. Tue, S. Baker, J.E. Bryant, G. Thwaites, J.J. Carrique-Mas, M.A. Rabaa);; Vietnam Academy of Sciences and Technology, Hanoi, Vietnam (N.T. Son);; Centers for Disease Control and Prevention Fort Collins, Colorado, USA (M. Kosoy);; University of Edinburgh, Edinburgh, Scotland, UK (M.E.J. Woolhouse, M.A. Rabaa);; London School of Hygiene and Tropical Medicine, London, UK (S. Baker);; Oxford University, Oxford, UK (S. Baker, J.E. Bryant, G. Thwaites, J.J. Carrique-Mas)

**Keywords:** Bartonella, bacteria, zoonoses, bats, reservoir species, diversity, Vietnam

**To the Editor:** To investigate bats as potential reservoirs for *Bartonella* spp. in Vietnam, we screened a range of bat species to determine the prevalence and genetic diversity of *Bartonella* spp. in bat populations in southern Vietnam. In a study of bat biodiversity in southern Vietnam, 60 bats were trapped at 6 sites in Dong Nai Culture and Nature Reserve and Cat Tien National Park, Vietnam, in May 2013. Bats were trapped by using mist nets and harp traps set at ground level, and were euthanized by using isoflurane (http://www.avma.org/KB/Policies/Documents/euthanasia.pdf) for cataloguing at the Vietnam Academy of Sciences and Technology, Hanoi. Blood specimens were collected by cardiac puncture, and external measurements were recorded. Bats were speciated according to morphology (*1*,*2*); trapped bats represented 10 species belonging to 5 genera. All species have been given a conservation status of least concern (http://www.iucnredlist.org/).

Total nucleic acid was extracted from blood samples by using the MagNApure automated nucleic acid extraction system (Roche, Basel, Switzerland). Extracted nucleic acid was subjected to conventional PCR to detect *Bartonella* spp. DNA by using primers specific for the citrate synthase A gene (*3*). A positive PCR result was determined by amplification of a 729-bp fragment. Twenty-one (35.0%) of 60 bat blood specimens had a result consistent with presence of *Bartonella* spp. (Table). Among insectivorous or carnivorous bats, *Bartonella* prevalence was 20 (45.5%) of 44 compared with 1 (6.2%) of 16 fruit-eating bats (χ^2^ = 6.3, p = 0.01). The prevalence of *Bartonella* spp. did not differ between sampling locations (Table) or by estimated age of the bat (determined by deviation above or below the median tibial length of each species); prevalence was 33.3% (9/27) in younger bats and 36.4% (12/33) in older bats.

**Table:** Prevalence of *Bartonella* spp. in bats from 2 sites in Dong Nai, Vietnam, 2013

Bat species	No. *Bartonella* spp.–positive bats/no. bats trapped (%)		
	Cat Tien National Park	Dong Nai Nature Reserve	Total
Cynopterus sphinx*	0/0	0/14	0/14 (0)
*Hipposideros armiger*†	2/6	0/0	2/6 (33.3)
*Hipposideros larvatui*†	3/5	0/0	3/5 (60)
Megaerops niphanae*	0/0	½	1/2 (50)
*Megaderma spasma*†	0/0	1/2	1/2 (50)
*Megaderma lyra*‡	1/1	0/0	1/1 (100)
*Rhinolophus acuminatus*†	0/0	9/17	9/17 (52.9)
*Rhinolophus chaseli*†	2/5	0/0	2/5 (40)
*Rhinolophus sinicus*†	0/3	2/4	2/7 (28.6)
*Rhinolophus luctus*†	0/1	0/0	0/1 (0)
Total	8/21 (38.1)	13/39 (33.3)	21/60 (35)

*Fruit-eating. †Insectivorous. ‡Carnivorous.

DNA sequences from the 21 PCR citrate synthase A gene amplicons (GenBank accession nos. KP100340–KP100360) were subjected to BLAST analysis (http://blast.ncbi.nlm.nih.gov/Blast.cgi) to assess sequence similarity. Potentially novel *Bartonella* phylogroups were identified as having <96% sequence similarity with all publicly available sequences in GenBank (*4*). The sequences were then manually aligned with those of a representative sample of *Bartonella* spp. and trimmed to the 327-nt region (positions 801–1127) commonly used for taxonomic classification (*4*). A neighbor-joining tree was constructed by using the Hasegawa–Kishino–Yano plus gamma model of nucleotide substitution in Geneious version 7.1.7 with 1,000 bootstrap replications (*5*).

Sequence analysis identified 10 distinct *Bartonella* phylogroups (I–X) among 21 *Bartonella-*positive blood samples from bats in Vietnam (online Technical Appendix, http://wwwnc.cdc.gov/EID/article/21/7/14-1760-Techapp1.pdf). Nine of these phylogroups showed <96% sequence similarity to all previously identified *Bartonella* sequences, suggesting they might belong to new *Bartonella* species. *Bartonella* spp. in *Rhinolophus* spp. bats were classified into phylogroups I, III, VIII, IX, and X. Phylogroups II and VII were detected in samples from *Hipposideros* spp. bats, and phylogroups IV and V were detected in *Megaderma* spp. bats. Phylogroup VI was detected only in a *Megaerops* spp. bat.

Although 9 lineages (I, III–X) were novel, phylogroup II was identified in 4 *Hipposideros* spp. bats and showed 96.3%–97.2% similarity to *Bartonella* spp. isolated from a bat fly found on a *Hipposideros* spp. host in Malaysia (GenBank accession no. JX416238). This similarity might suggest widespread distribution of this *Bartonella* spp. lineage in *Hipposideros* spp. bats or their ectoparasites in Southeast Asia. Additional genetic characterization of strains is needed to determine whether any of these novel phylogroups represent new species and to investigate their evolutionary and ecological relationships with other *Bartonella* spp. identified in Vietnam and elsewhere.

The primary observation in this study was detection of *Bartonella* spp. (by DNA amplification) in bats in southern Vietnam at a prevalence of 35.0%, which is comparable with that reported in Kenya (30.2%) and Guatemala (33.0%) (Table) (*6*,*7*). However, the use of conventional PCR in this study might underestimate the true prevalence.

Although high prevalences have been proposed to be caused by persistent infection of bats with *Bartonella* spp., our findings indicate no increase in prevalence by age of bat, which would be expected if persistent infection were common. This finding, and detection of multiple lineages infecting individual bat species, may instead reflect high levels of transmission within and between bat species caused by crowded roosting areas and sharing of roosts by multiple species. This behavior provides opportunities for transmission of *Bartonella* bacteria or exchange of infected ectoparasites, such as *Cyclopodia* spp. (*8*), although the precise roles of these 2 processes are unknown.

Although no human cases of *Bartonella* spp. infection have been reported in Vietnam, *Bartonella* spp. have been identified in febrile humans elsewhere in Southeast Asia (*9*) and are also common in rats in southern Vietnam (*10*). Because close contact with bats (i.e., through manure farming and consumption of bat meat) and potential arthropod vectors (i.e., through handling and consumption of fruit) is common in parts of Vietnam, targeted screening of bats and their human contacts might improve our understanding of the zoonotic potential of these bacteria and their potential effect on public health.

**Technical Appendix.** Neighbor-joining phylogeny of a 327-nt region of the citrate synthase A gene of 21 *Bartonella* spp. detected in bats in southern Vietnam and a sample of diverse *Bartonella* spp. causing infections in animals and humans globally. *Brucella abortus* (AE017223) is included as an outgroup. Bootstrap support values are shown for nodes with >80% support. Taxa names for sequences determined in this study are indicated in bold italics, phylogroups are indicated in parentheses (I–X), and bat species are indicated after phylogroups.
