# Supplementary figures and images for: Diversity of Bartonella spp. in Bats, Southern Vietnam
Source: Emerg Infect Dis. 2015 Jul;21(7):1266–7. doi: 10.3201/eid2107.141760 (PMC4480386; doi:10.3201/eid2107.141760)

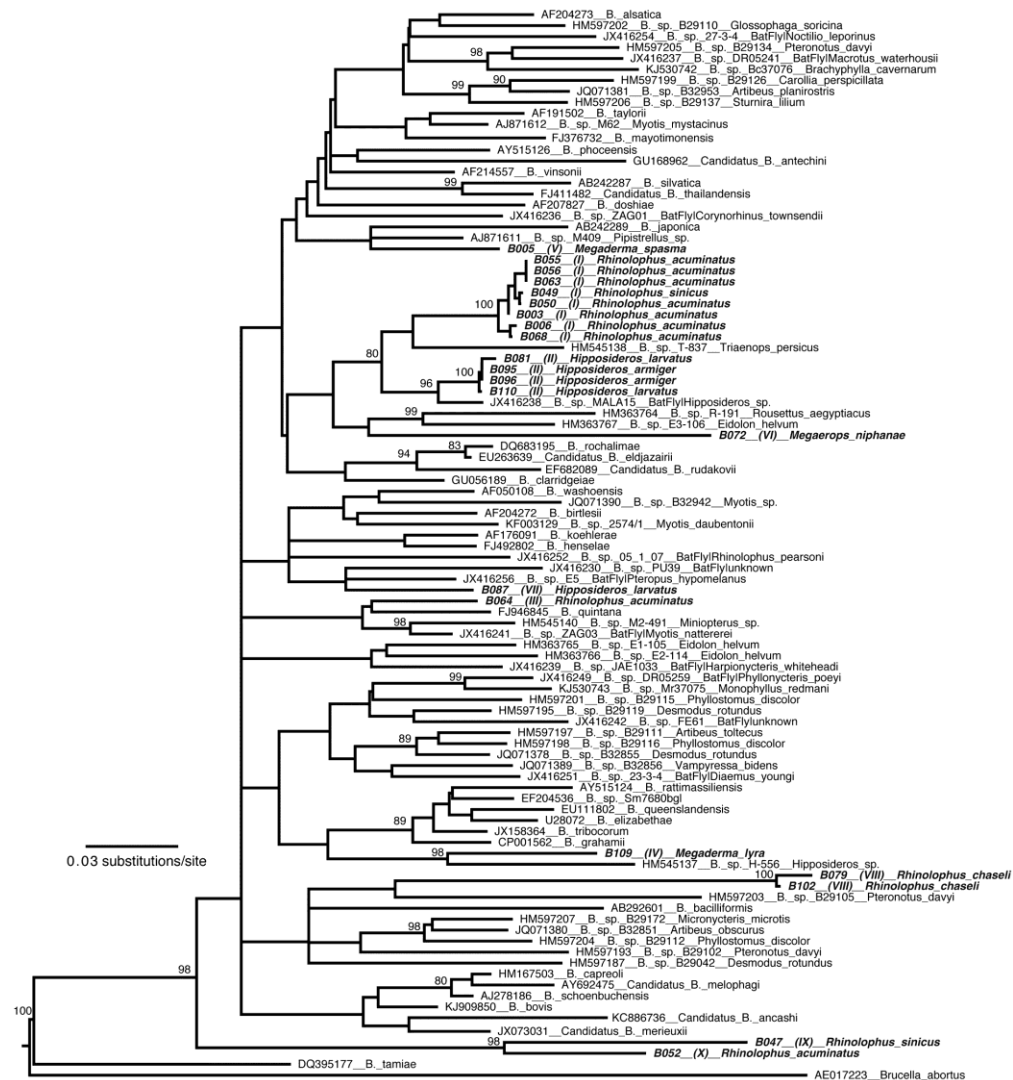

Supplement: Supplementary file 1 — Technical Appendix. Neighbor-joining phylogeny of a 327-nt region of the citrate synthase A gene of 21 Bartonella spp. detected in bats in southern Vietnam and a sample of diverse Bartonella spp. causing infections in animals and humans globally. Brucella abortus (AE017223) is included as an outgroup. Bootstrap support values are shown for nodes with >80% support. Taxa names for sequences determined in this study are indicated in bold italics, phylogroups are indicated in parentheses (I–X), and bat species are indicated after phylogroups. [file 14-1760-Techapp-s1.pdf]
